# Dual inhibition of α_v_β_6_ and α_v_β_1_ reduces fibrogenesis in lung tissue explants from patients with IPF

**DOI:** 10.1186/s12931-021-01863-0

**Published:** 2021-10-19

**Authors:** Martin L. Decaris, Johanna R. Schaub, Chun Chen, Jacob Cha, Gail G. Lee, Megi Rexhepaj, Steve S. Ho, Vikram Rao, Megan M. Marlow, Prerna Kotak, Erine H. Budi, Lisa Hooi, Jianfeng Wu, Marina Fridlib, Shamra P. Martin, Shaoyi Huang, Ming Chen, Manuel Muñoz, Timothy F. Hom, Paul J. Wolters, Tushar J. Desai, Fernando Rock, Katerina Leftheris, David J. Morgans, Eve-Irene Lepist, Patrick Andre, Eric A. Lefebvre, Scott M. Turner

**Affiliations:** 1 Pliant Therapeutics, South San Francisco, CA USA; 2 Department of Medicine, University of California, San Francisco, CA USA; 3 Department of Medicine, Stanford University, Stanford, CA USA; 4 Present Address: Acceleron Pharma, Cambridge, MA USA; 5 Present Address: Maze Therapeutics, South San Francisco, CA USA

**Keywords:** Transforming growth factor-β, Precision-cut lung slice, Antifibrotic, α_v_ integrin, PLN-74809

## Abstract

**Rationale:**

α_v_ integrins, key regulators of transforming growth factor-β activation and fibrogenesis in in vivo models of pulmonary fibrosis, are expressed on abnormal epithelial cells (α_v_β_6_) and fibroblasts (α_v_β_1_) in fibrotic lungs.

**Objectives:**

We evaluated multiple α_v_ integrin inhibition strategies to assess which most effectively reduced fibrogenesis in explanted lung tissue from patients with idiopathic pulmonary fibrosis.

**Methods:**

Selective α_v_β_6_ and α_v_β_1_, dual α_v_β_6_/α_v_β_1_, and multi-α_v_ integrin inhibitors were characterized for potency, selectivity, and functional activity by ligand binding, cell adhesion, and transforming growth factor-β cell activation assays. Precision-cut lung slices generated from lung explants from patients with idiopathic pulmonary fibrosis or bleomycin-challenged mouse lungs were treated with integrin inhibitors or standard-of-care drugs (nintedanib or pirfenidone) and analyzed for changes in fibrotic gene expression or TGF-β signaling. Bleomycin-challenged mice treated with dual α_v_β_6_/α_v_β_1_ integrin inhibitor, PLN-74809, were assessed for changes in pulmonary collagen deposition and Smad3 phosphorylation.

**Measurements and main results:**

Inhibition of integrins α_v_β_6_ and α_v_β_1_ was additive in reducing type I collagen gene expression in explanted lung tissue slices from patients with idiopathic pulmonary fibrosis. These data were replicated in fibrotic mouse lung tissue, with no added benefit observed from inhibition of additional α_v_ integrins. Antifibrotic efficacy of dual α_v_β_6_/α_v_β_1_ integrin inhibitor PLN-74809 was confirmed in vivo, where dose-dependent inhibition of pulmonary Smad3 phosphorylation and collagen deposition was observed. PLN-74809 also, more potently, reduced collagen gene expression in fibrotic human and mouse lung slices than clinically relevant concentrations of nintedanib or pirfenidone.

**Conclusions:**

In the fibrotic lung, dual inhibition of integrins α_v_β_6_ and α_v_β_1_ offers the optimal approach for blocking fibrogenesis resulting from integrin-mediated activation of transforming growth factor-β.

**Supplementary Information:**

The online version contains supplementary material available at 10.1186/s12931-021-01863-0.

## Background

Idiopathic pulmonary fibrosis (IPF) is a fatal disease characterized by progressive lung scarring, impaired oxygen diffusion, exertional dyspnea, and a mean life expectancy of < 4 years [1, 2]. Current standard-of-care drugs for IPF, nintedanib and pirfenidone, have modest effects on pulmonary function and life expectancy; however, neither halt disease progression or consistently improve quality of life [3–5]*.* Improved strategies for treating IPF are required.

The pathobiology of IPF, although incompletely understood, is thought to initiate from chronic injury to and/or aging of the alveolar epithelium, resulting in an aberrant wound healing response and the sustained production of pro-inflammatory and profibrotic factors [1, 2]. This ultimately leads to the activation and differentiation of perivascular and interstitial mesenchymal cells into myofibroblasts, the primary cell population responsible for pulmonary fibrogenesis (e.g. collagen synthesis). Elevated transforming growth factor-β (TGF-β) signaling is a hallmark of IPF, promoting fibroblast-to-myofibroblast transition, collagen gene expression, and the deposition of scar tissue which impairs pulmonary function [6, 7]. Pharmacological inhibition of TGF-β therefore offers a promising approach for treating IPF. However, because TGF-β also regulates many important homeostatic functions throughout the body, its systemic inhibition may result in toxicities and a targeted approach for TGF-β inhibition is desired [8–12].

TGF-β is secreted in a latent (inactive) form, requiring extracellular enzymatic or mechanically induced activation to engage cell surface receptors [9, 11]. α_v_ integrins (α_v_β_1_, α_v_β_3_, α_v_β_5_, α_v_β_6_, and α_v_β_8_)—five heterodimeric transmembrane proteins capable of transducing mechanical force between cells and the ECM—have been proposed as key mediators of TGF-β activation in fibrosis. α_v_ integrins are upregulated in fibrotic tissues and can induce activation of two of the three TGF-β isoforms (TGF-β_1_ and TGF-β_3_) through binding to the Arg-Gly-Asp (RGD) sequence present in the latent TGF-β complex [11, 13]. Targeting α_v_ integrins, therefore, represents an appealing strategy for restricting TGF-β signaling inhibition to fibrotic tissues.

To date, it remains unclear which subset of α_v_ integrins is optimal to target in fibrotic human lungs. Elevated α_v_β_6_ levels in lung epithelium of patients with IPF correlate with disease progression rate, and integrin subunit β_6_ (ITGB6) knockout mice are protected from bleomycin-induced lung fibrosis [14–16]. Both pharmacological inhibition of α_v_β_1_, expressed primarily on fibroblasts, and conditional knockdown of β_8_ in fibroblasts, have also been shown to improve outcome in mouse models of lung and airway fibrosis [17–20]. While α_v_β_3_ and α_v_β_5_ have been implicated in profibrotic mechanosignaling by myofibroblasts, dual α_v_β_3_/α_v_β_5_ knockout mice are not protected from bleomycin-induced lung fibrosis, and α_v_β_3_ antagonists exacerbated liver fibrosis in mouse models [17, 21–25].

Previous studies demonstrating antifibrotic effects from α_v_ integrin inhibition [13, 14, 20] have largely been limited to the bleomycin mouse model, which does not precisely replicate human disease [26, 27]. Precision-cut lung slices (PCLSs) generated from lung explants of patients with IPF collected at time of transplant, on the other hand, offer a physiologically relevant ex vivo platform to study fibrosis, preserving the diverse cellular and ECM composition and architecture of fibrotic human lungs [28–30].

Using a well-characterized set of small-molecule and antibody-based integrin inhibitors, we evaluated multiple α_v_ integrin inhibition strategies to assess which most effectively reduced TGF-β signaling and fibrogenic gene expression in human PCLSs.

## Methods

Additional details for the methods described below are provided in an Additional file 1.

### Human lung tissue

Tissue samples from patients with IPF were acquired at the time of lung transplantation. Written informed consent was obtained from all subjects, and the study was approved by the University of California Committee on Human Research (San Francisco, CA, USA) or Stanford University Institutional Review Board (Stanford, CA, USA). Rejected donor lung tissues were acquired from the University of California or Promethera Biosciences (Durham, NC, USA) with appropriate authorizations.

### Cell culture and reagents

Details for primary cells, cell lines, culture conditions, and reagents are provided in Additional file 1.

### Quantitation of α_v_β_1_ integrin and Smad2/3 phosphorylation levels

Integrin α_v_β_1_ protein levels and Smad phosphorylation levels in tissues or cells were quantified by the Meso Scale Discovery custom electrochemiluminescence assay.

### Integrin ligand-binding assays

Integrin ligand-binding assays were performed similar to that previously described [31].

### TGF-β co-culture assays

α_v_β_6_- or α_v_β_1_-expressing cells were co-cultured with mink lung epithelial cells and small-molecule integrin inhibitors to determine potency for blocking TGF-β activation, similar to that previously described [20, 32].

### Primary cell latency-associated peptide (LAP) adhesion assays

Adhesion of primary human lung cells to LAP was quantified using the xCELLigence RTCA MP instrument (ACEA Biosciences; San Diego, CA, USA) via cell impedance measurements similar to that previously described [33].

### PCLS preparation, culture, and gene expression analysis

PCLSs were generated from explanted lung tissue from patients with IPF and cultured as previously described [30, 34–38]. Mouse PCLSs were generated and cultured as previously described [39]. Messenger ribonucleic acid (mRNA) levels in lysates prepared from snap-frozen tissue slices were quantified via NanoString PlexSet reagents and nCounter SPRINT Profiler (NanoString; Seattle, WA, USA; Additional file 1: Table S2) or TaqMan primers/probes on a Bio-Rad CFX96 thermocycler (Bio-Rad; Hercules, CA, USA; Additional file 1: Table S3).

### In vivo bleomycin model and tissue collection

C57BL/6 mice (Charles River; Hollister, CA, USA) were administered 3 units/kg bleomycin (Teva; Toronto, ON, Canada) or vehicle (water) via oropharyngeal aspiration. Lung tissue and bronchioalveolar lavage (BAL) cells were collected after 14 or 21 days to evaluate Smad phosphorylation and collagen deposition.

### Measurement of plasma PLN-74809 concentrations

Plasma concentration of PLN-74809, a dual α_v_β_6_/α_v_β_1_ inhibitor, was measured by liquid chromatography with tandem mass spectrometry.

### Total lung hydroxyproline (OHP) content and fractional synthesis rate

OHP levels and fractional synthesis rates were determined similar to that previously described [40, 41].

### Second harmonic generation (SHG) imaging

Formalin-fixed, paraffin-embedded mouse lung tissue sections underwent SHG imaging to characterize the quantity and quality of fibrillar collagen deposition using methods similar to that previously described [42].

### Statistics

For comparison of three or more groups within a single experiment, data were compared via one-way analysis of variance (ANOVA) with Dunnett’s multiple comparisons test performed between each test group and the vehicle-treated group. For comparison of three or more groups across multiple PCLS experiments (i.e. multiple donor tissues), donor-normalized data were compared via repeated measures, one-way ANOVA, or a mixed-effects model with Dunnett’s multiple comparisons test performed between each test group and the vehicle-treated group.

## Results

### Potency and selectivity of small-molecule integrin inhibitors

The potency and selectivity of small-molecule α_v_ integrin inhibitors were profiled using human integrin ligand-binding assays (Table 1). PLN-74809, a dual α_v_β_6_/α_v_β_1_ inhibitor, was roughly equipotent for inhibition of α_v_β_6_ and α_v_β_1_ (respective 50% inhibitory concentration [IC_50_] values of 5.7 nM and 3.4 nM), with ≥ 445-fold selectivity over other α_v_ integrins. An α_v_β_1_-specific small-molecule inhibitor, Compound A, was determined to have an IC_50_ of 2.3 nM for α_v_β_1_ with 22-fold selectivity over α_v_β_6_ and > 50-fold selectivity over other α_v_ integrins. GSK3008348 and CWHM-12, α_v_ integrin inhibitors previously described in the literature [13, 43, 44], were determined to be less selective for α_v_β_6_ and α_v_β_1_ than PLN-74809 and Compound A, respectively. An α_v_β_6_-specific blocking antibody, 3G9, with previously described selectivity profile [45] was found to inhibit α_v_β_6_-ligand binding with an IC_50_ of 7.34 nM.

**Table 1:** Ligand-binding assay IC_50_ and TGF-β activation assay IC_50_ for small molecule integrin inhibitors

Compound A (α_v_β_1_ inhibitor)			PLN-74809 (dual α_v_β_6_/α_v_β_1_)		
Integrin	Ligand binding IC_50_ (nM)	Fold selectivity (vs α_v_β_1_)	Integrin	Ligand binding IC_50_ (nM)	Fold selectivity (vs α_v_β_6_)
α_v_β_1_	2.3	1×	α_v_β_6_	5.7	1×
α_v_β_6_	49.8	22×	α_v_β_1_	3.4	0.6×
α_v_β_3_	116.4	51×	α_v_β_3_	> 10,000	> 1754×
α_v_β_5_	504	221×	α_v_β_5_	6989	1226×
α_v_β_8_	2933	1286×	α_v_β_8_	2539	445×

Integrin	TGF-β activation IC_50_ (nM)	Fold selectivity (vs α_v_β_1_)	Integrin	TGF-β activation IC_50_ (nM)	Fold selectivity (vs α_v_β_6_)
α_v_β_1_	7.9	1×	α_v_β_6_	29.8	1×
α_v_β_6_	6047	765×	α_v_β_1_	19.2	0.6×

CWHM-12 (multi-α_v_ inhibitor)			GSK3008348 (multi-α_v_ inhibitor)		
Integrin	Ligand binding IC_50_ (nM)	Fold selectivity (vs α_v_β_1_)	Integrin	Ligand binding IC_50_ (nM)	Fold selectivity (vs α_v_β_6_)
α_v_β_1_	1.3	1×	α_v_β_6_	3.4	1×
α_v_β_6_	6.1	4.7×	α_v_β_1_	4.0	1.2×
α_v_β_3_	0.7	0.5×	α_v_β_3_	299	89×
α_v_β_5_	5.3	4.1×	α_v_β_5_	23.5	7.0×
α_v_β_8_	7.3	5.6×	α_v_β_8_	6.8	2.0×

Integrin	TGF-β activation IC_50_ (nM)	Fold selectivity (vs α_v_β_1_)	Integrin	TGF-β activation IC_50_ (nM)	Fold selectivity (vs α_v_β_6_)
α_v_β_1_	2	1×	α_v_β_6_	4.7	1×
α_v_β_6_	318.0	159×	α_v_β_1_	17.4	3.7×

IC_50_: 50% inhibitory concentration; TGF-β: Transforming growth factor-β

Potency and selectivity of small-molecule α_v_ integrin inhibitors for blocking latent TGF-β activation by α_v_β_6_ and α_v_β_1_ was also evaluated in co-culture assays combining integrin α_v_β_6_- or α_v_β_1_-expressing cells with a TGF-β-sensitive reporter cell line (Table 1).

PLN-74809 inhibited α_v_β_6_- and α_v_β_1_-induced TGF-β activation with IC_50_ values of 29.8 nM and 19.2 nM, respectively. Selective α_v_β_1_ inhibitor, Compound A, blocked α_v_β_1_- and α_v_β_6_-induced TGF-β activation with IC_50_ values of 7.9 nM and 6047 nM, respectively, demonstrating > 750-fold functional selectivity for α_v_β_1_ over α_v_β_6_, despite the 22-fold biochemical selectivity. α_v_β_6_-specific blocking antibody, 3G9, inhibited α_v_β_6_-induced TGF-β activation with an IC_50_ value of 0.19 nM.

### Antifibrogenic effects of integrin inhibitors in PCLSs prepared from lung tissue from patients with IPF

**Fig. 1:**
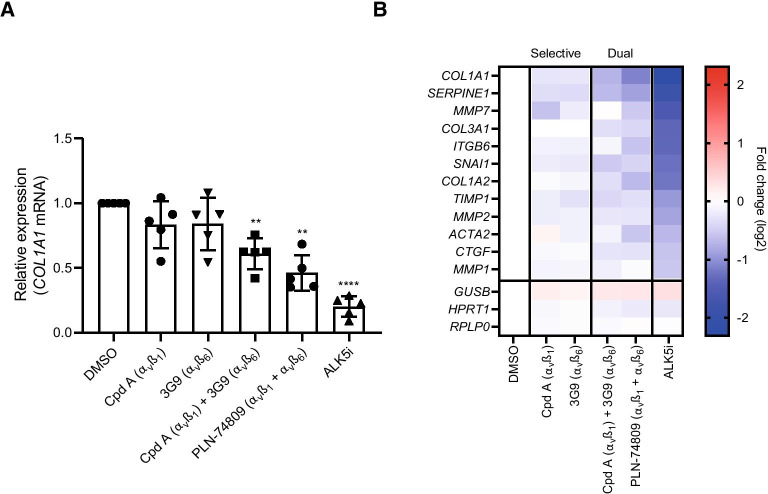
Effect of α_v_β_1_-selective inhibition (Compound A), α_v_β_6_-selective inhibition (3G9), and dual α_v_β_6_/α_v_β_1_ inhibition (PLN-74809 or Compound A + 3G9) on **A** *COL1A1* mRNA expression and **B** expression of additional fibrosis-related genes following 7-day culture of PCLSs prepared from lung explants from patients with IPF. Data represent mean (± SD) of 4–5 independent IPF tissues with ≥ 3 slices analyzed per patient tissue. Each symbol within a group represents an individual patient lung, with treatment effects normalized to vehicle. Data in **A** and **B** were generated from the same samples. Compound A = 471 nM; 3G9 = 0.5 µg/ml; PLN-74809 = 1.82 µM; TGF-β type I receptor inhibitor (ALK5i [R 268712]) = 1 µM. ALK5i was used as a positive control to confirm TGF-β-driven collagen expression within lung slices. Concentrations selected for integrin inhibitors were ≥ 10 × IC_50_, determined to inhibit latent TGF-β activation by α_v_β_1_ or α_v_β_6_ in cell-based assays (see Table 1). **P < 0.01 vs DMSO; ****P < 0.0001 vs DMSO. *ACTA2*: α-smooth muscle actin 2; ALK5i: Activin receptor-like kinase 5 inhibitor; *COL1A1*: Collagen type I alpha I; *COL1A2*: Collagen type I alpha II; *COL3A1*: Collagen type III alpha I; Cpd A: Compound A; CTGF: Connective tissue growth factor; DMSO: Dimethyl sulfoxide; *GUSB*: Glucuronidase β; IC_50_: 50% inhibitory concentration; *HPRT1*: Hypoxanthine phosphoribosyltransferase 1; IPF: Idiopathic pulmonary fibrosis; *ITGB6*: Integrin subunit β 6; *MMP1*: Matrix metalloproteinase 1; *MMP2*: Matrix metalloproteinase 2; *MMP7*: Matrix metalloproteinase 7; mRNA: Messenger ribonucleic acid; PCLS: Precision-cut lung slice; *RPLP0*: Ribosomal lateral stalk subunit P0; SD: Standard deviation; *SERPINE1*: Serpin family E member 1; *SNAI1*: Snail family transcriptional repressor 1; TGF-β: Transforming growth factor-β; *TIMP1*: Tissue inhibitor of metalloproteinase 1

PCLSs prepared from explanted lung tissue from patients with IPF (*n* = 5–7 individual patients; *n* ≥ 3 slices per patient/treatment) were treated with integrin inhibitors or vehicle for 7 days prior to fibrogenic gene expression analysis. Donor history for human lung samples is listed in Additional file 1: Table S1. Slices from all lungs had good viability following ex vivo culture (Additional file 1: Fig. S1A). Following a 7-day incubation, dual α_v_β_6_/α_v_β_1_ inhibitor, PLN-74809, or a combination of α_v_β_6_-inhibiting antibody, 3G9, and α_v_β_1_-small-molecule inhibitor, Compound A, significantly reduced collagen type I alpha I (*COL1A1*) mRNA expression by 54% and 39%, respectively (Fig. 1A; *P* < 0.01). Inhibition of α_v_β_6_ alone with 3G9, or α_v_β_1_ alone with Compound A, did not significantly reduce *COL1A1* mRNA expression in PCLSs from the same individuals. Analysis of additional fibrosis-related genes showed a similar effect, with dual α_v_β_6_/α_v_β_1_ inhibition resulting in a greater reduction in expression of α-smooth muscle actin 2 (*ACTA2*; 33% decrease, *P* < 0.01) and plasminogen activator inhibitor 1 (serpin family E member 1 [*SERPINE1*]; 45% decrease, *P* < 0.01) than inhibition of either integrin alone (Fig. 1B). Integrin inhibitors were tested at concentrations ≥ 10 × IC_50_ for blocking α_v_β_6_ and/or α_v_β_1_ integrin-mediated TGF-β activation as described in Table 1. A potent inhibitor of TGF-β receptor I (activin receptor-like kinase 5 [ALK5]), R-268712, was also used as a positive control (100 × reported IC_50_). ALK5 inhibition blocked collagen gene expression by ~ 80% (*P* < 0.0001), confirming TGF-β-driven collagen gene expression in the PCLSs.

Follow-up analysis of PCLSs (*n* = 3 individual patients) treated with dual α_v_β_6_/α_v_β_1_ inhibitor PLN-74809 for 7 days showed an approximately 50% reduction in Smad2 phosphorylation, a marker of canonical TGF-β signaling, consistent with a TGF-β-inhibitory mechanism of action (Additional file 1: Fig. S1B; *P* < 0.001). PCLSs generated from a single patient with IPF used to perform a dose titration of PLN-74809 indicated that concentrations as low as 2 nM were sufficient to significantly reduce *COL1A1* expression (Additional file 1: Fig. S1C; *P* < 0.0001).

### Antifibrogenic effects of integrin inhibitors in PCLSs prepared from fibrotic mouse tissue

Due to a scarcity of explanted tissue from patients with IPF, the bleomycin mouse model of pulmonary fibrosis was utilized as a supplemental source of fibrotic lung tissue for PCLS experiments. Additive antifibrotic effects of α_v_β_6_ and α_v_β_1_ inhibition in PCLSs prepared from bleomycin-challenged mouse lungs were examined to assess translatability of experimental results between mouse and human lung tissue.

**Fig. 2:**
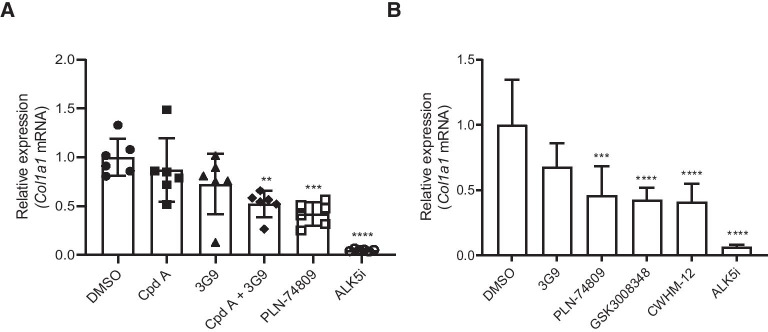
Effect of selective α_v_β_6_ or α_v_β_1_, dual α_v_β_6_/α_v_β_1_, or multi-α_v_ inhibition on *Col1a1* expression in **A** PCLSs generated from chronic bleomycin-challenged mouse lungs and **B** PCLSs generated from acute bleomycin-challenged mouse lungs. **A** Compound A (α_v_β_1_-selective inhibitor) = 471 nM; 3G9 (α_v_β_6_-selective inhibitor) = 0.5 µg/ml; PLN-74809 (dual α_v_β_6_/α_v_β_1_ inhibitor) = 1.82 µM; ALK5i (R 268,712) = 1 µM. Data are mean (± SD) of a single slice from *n* = 6 mouse lungs. Symbols represent results for individual animals. Culture and treatment were for 7 days. Treatment effects were normalized to average DMSO control. Concentrations selected for integrin inhibitors were ≥ 10 × IC_50_ determined to inhibit latent TGF-β activation by α_v_β_1_ or α_v_β_6_ (see Table 1). **B** 3G9 (α_v_β_6_-selective inhibitor) = 1 µg/ml; PLN-74809 (dual α_v_β_6_/α_v_β_1_ inhibitor) = 200 nM; GSK3008348 (multi-α_v_ inhibitor) and CWHM-12 (multi-α_v_ inhibitor) = 1 µM. Data are mean (± SD) of a single slice from *n* = 5–6 mouse lungs. Culture and treatment were for 3 days. Treatment effects were normalized to DMSO control. **P < 0.01 vs DMSO; ***P < 0.001 vs DMSO; ****P < 0.0001 vs DMSO. ALK5i: Activin receptor-like kinase 5 inhibitor; *Col1a1*: Collagen type I alpha I; Cpd A: Compound A; DMSO: Dimethyl sulfoxide; IC_50_: 50% inhibitory concentration; mRNA: Messenger ribonucleic acid; PCLS: Precision-cut lung slice; SD: standard deviation; TGF-β: transforming growth factor-β

Following 3 days of treatment, dual α_v_β_6_/α_v_β_1_ inhibitor PLN-74809 dose-dependently reduced *Col1a1* mRNA expression in PCLSs prepared from fibrotic mouse lungs by up to 71%, with a significant reduction observed at concentrations as low as 16 nM (Additional file 1: Fig. S2; *P* < 0.05). Similar to our observation in human PCLSs, dual inhibition of α_v_β_6_/α_v_β_1_ for 7 days with PLN-74809 or a combination of α_v_β_6_ inhibitor, 3G9, and α_v_β_1_ inhibitor, Compound A, was also effective at significantly reducing *Col1a1* mRNA expression in PCLSs prepared from fibrotic mouse lungs, whereas treatment with 3G9 or Compound A alone was not (Fig. 2A). Dual α_v_β_6_/α_v_β_1_ inhibition with PLN-74809 (200 nM) was also shown to inhibit collagen mRNA expression to virtually the same degree as less selective multi-α_v_ integrin inhibitors, GSK3008348 (1000 nM) and CWHM-12 (1000 nM), following 3 days of treatment in PCLSs prepared from acutely injured mouse lungs, indicative of no additional benefit from inhibition of integrins α_v_β_3_, α_v_β_5_, or α_v_β_8_ in reducing fibrogenesis (Fig. 2B).

### Characterization of α_v_β_1_ in fibrotic lung tissue

**Fig. 3:**
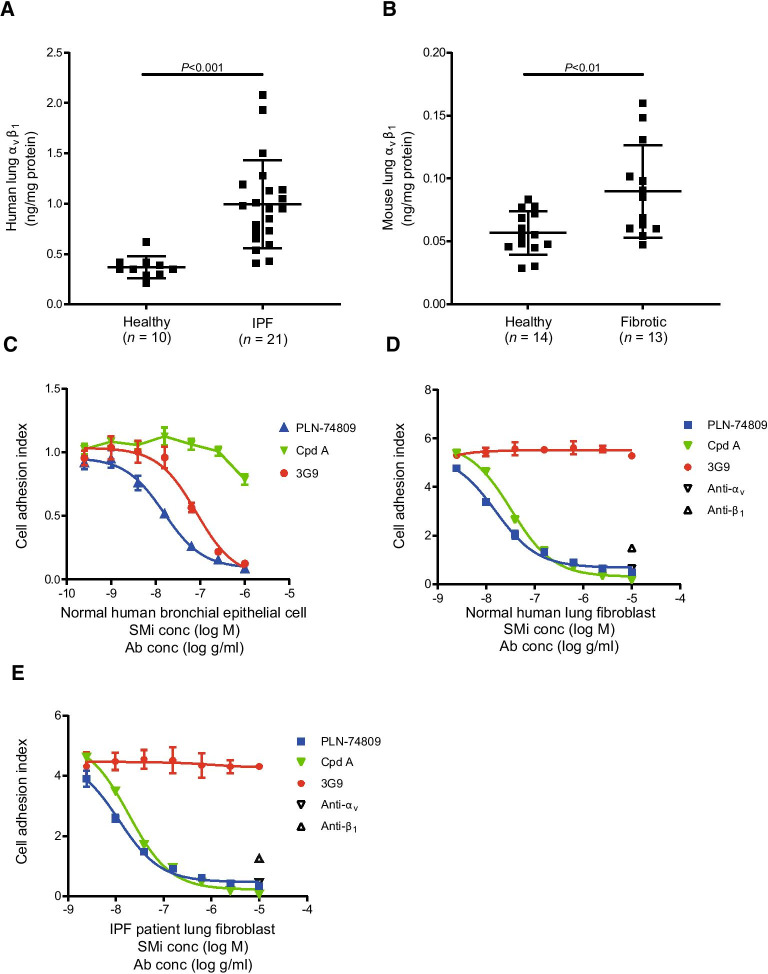
Mean (± SD) α_v_β_1_ protein levels measured in **A** lung tissue from healthy subjects vs patients with IPF and **B** healthy vs fibrotic mouse lung tissue (21 days post-bleomycin challenge). Efficacy of integrin inhibitors of α_v_β_6_ (3G9), α_v_β_1_ (Compound A), or α_v_β_6_/α_v_β_1_ (PLN-74809) at blocking (**C**) normal human bronchial epithelial cell, **D** normal human lung fibroblast, and **E** IPF lung fibroblast adhesion to TGF-β LAP as determined by cell impedance assay. One representative donor cell plotted in (**C**–**E**) (mean [± SD] for *n* = 3 replicate measurements). Pan-α_v_ integrin- and pan-β_1_ integrin-inhibiting antibodies were also used to demonstrate α_v_β_1_-mediated adhesion of normal and IPF lung fibroblasts to LAP (**D**, **E**). Ab: antibody; conc: concentration; Cpd A: Compound A; IPF: idiopathic pulmonary fibrosis; LAP: latency-associated peptide; SD: standard deviation; SMi: small-molecule inhibitor; TGF-β: transforming growth factor-β

While upregulation of α_v_β_6_ in fibrotic human lung tissue has previously been described, little is known about α_v_β_1_ protein levels in this setting. We therefore quantified α_v_β_1_ protein levels in healthy and fibrotic human lung tissue via custom electrochemiluminescence assay. Donor history for human lung samples is listed in Additional file 1: Table S1. Mean α_v_β_1_ protein levels were elevated by 2.7-fold (Fig. 3A; *P* < 0.001) in lung tissue from patients with IPF (*n* = 21) compared with healthy subjects (*n* = 10). A similar approach was used to assess α_v_β_1_ levels in fibrotic mouse lungs 21 days following bleomycin challenge. Mean α_v_β_1_ protein levels were elevated by 1.6-fold (Fig. 3B; *P* < 0.01) in bleomycin-challenged lung tissue compared to healthy mouse lung tissue.

### Inhibition of primary lung epithelial cell and fibroblast adhesion to LAP

Dual α_v_β_6_/α_v_β_1_ inhibitor, PLN-74809, was tested alongside specific inhibitors of α_v_β_6_ (3G9) and α_v_β_1_ (Compound A) to confirm the respective role of these integrins in mediating primary human lung epithelial cell (*n* = 2 individual healthy donors) and primary human lung fibroblast (HLF; *n* = 3 individual healthy donors and *n* = 4 donors with IPF) adhesion to LAP. PLN-74809 and 3G9 fully inhibited α_v_β_6_ integrin-mediated adhesion to LAP by normal human bronchial epithelial cells with IC_50_ values of 39.3 nM and 0.368 nM (57.9 ng/ml), respectively (Fig. 3C). α_v_β_1_-selective inhibitor, Compound A, was unable to effectively block normal human bronchial epithelial cell adhesion to LAP at the concentrations tested (IC_50_ > 1000 nM; Fig. 3C). PLN-74809, but not 3G9, was also able to block integrin-mediated adhesion to LAP by HLFs isolated from normal or fibrotic lung tissue, with IC_50_ values of 15.7 nM and 23.5 nM, respectively (Fig. 3D, E). α_v_β_1_-selective inhibitor, Compound A, also blocked normal and IPF HLFs from adhering to LAP with IC_50_ values of 26.1 nM and 24.4 nM, respectively. Individually, pan-α_v_ integrin- and pan-β_1_ integrin-blocking antibodies also inhibited HLF adhesion to LAP, indicating that α_v_β_1_ specifically, not additional α_v_- or β_1_-containing integrins, drives this cell–ligand interaction.

### Antifibrotic effects of dual α_v_β_6_/α_v_β_1_ inhibition in the bleomycin mouse model

**Fig. 4:**
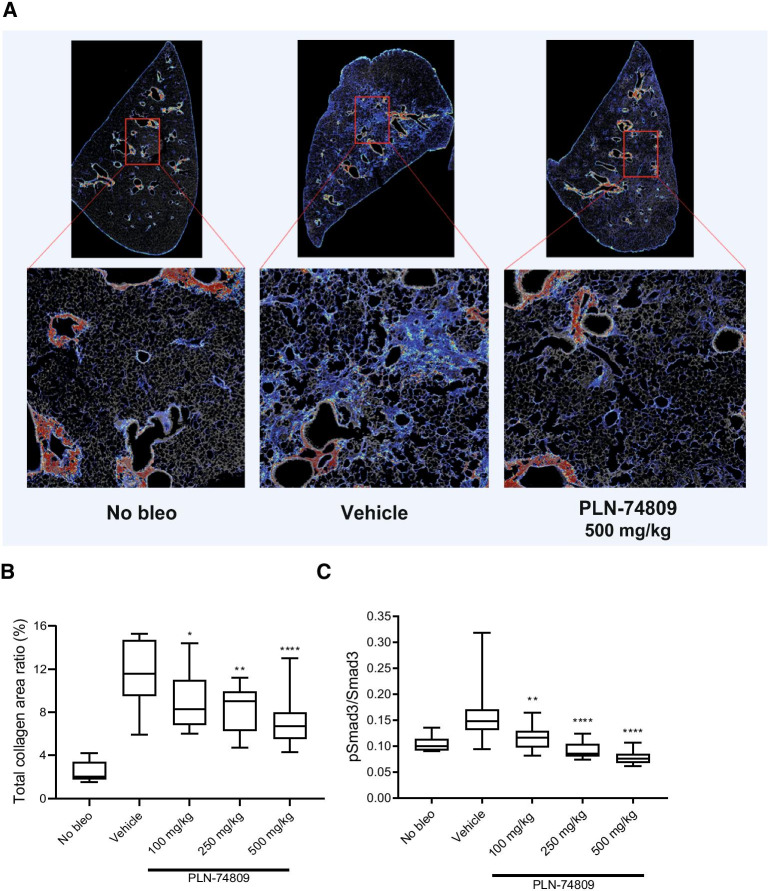
Pulmonary collagen deposition and Smad3 phosphorylation in bleomycin-challenged mice receiving dual α_v_β_6_/α_v_β_1_ inhibitor. **A** SHG microscopy images of interstitial collagen deposition (blue) in lung tissue sections collected from sham-challenged or bleomycin-challenged mice (vehicle or 500 mg/kg PLN-74809). **B** Morphometric analysis of interstitial fibrillar collagen deposition from SHG imaging, and **C** pSmad3/Smad3 ratio in lung tissue from bleomycin-challenged mice treated with PLN-74809 (100, 250, and 500 mg/kg) or vehicle was compared to sham-challenged mice. PLN-74809 was dosed orally (100–500 mg/kg BID) in mice from 7 to 21 days post-bleomycin-induced lung injury. **A** Representative images show both fibrotic interstitial fine collagen fibers (blue) and denser normal structural collagens surrounding airways (red). **B**, **C** Data presented as box and whisker plot with minimum, 25th, 50th, 75th percentile, and maximum values indicated. *P < 0.05; **P < 0.01; ****P < 0.0001. BID: twice daily; Bleo: bleomycin; PBS: phosphate-buffered saline; pSmad3: phosphorylated Smad3; SHG: second harmonic generation

While dual α_v_β_6_/α_v_β_1_ inhibition significantly reduced markers of fibrogenesis (TGF-β signaling and collagen gene expression) in IPF PCLSs, this model system is not conducive to measuring changes in collagen protein deposition, a process which occurs at a slower pace. We therefore investigated the effects of dual α_v_β_6_/α_v_β_1_ inhibition on collagen deposition in vivo using the standard bleomycin mouse model of pulmonary fibrosis. Dual α_v_β_6_/α_v_β_1_ inhibitor, PLN-74809, was dosed orally (100, 250, and 500 mg/kg twice daily [BID]) from Day 7 to Day 21 following bleomycin challenge. Morphometric analysis of lung tissue sections following SHG imaging showed a dose-dependent, significant reduction in interstitial fibrillar collagen deposition in bleomycin-challenged mice receiving PLN-74809 compared with vehicle (Fig. 4A, B). PLN-74809 also dose-dependently blocked Smad3 phosphorylation (Fig. 4C), consistent with inhibition of latent TGF-β activation. Total pulmonary hydroxyproline levels and deposition rate—the latter being determined by measuring ^2^H incorporation into newly synthesized hydroxyproline following stable isotope labeling of mice with ^2^H_2_O—were also dose-dependently reduced by PLN-74809 when compared with vehicle-treated mice (Additional file 1: Fig. S3A, B).

**Fig. 5:**
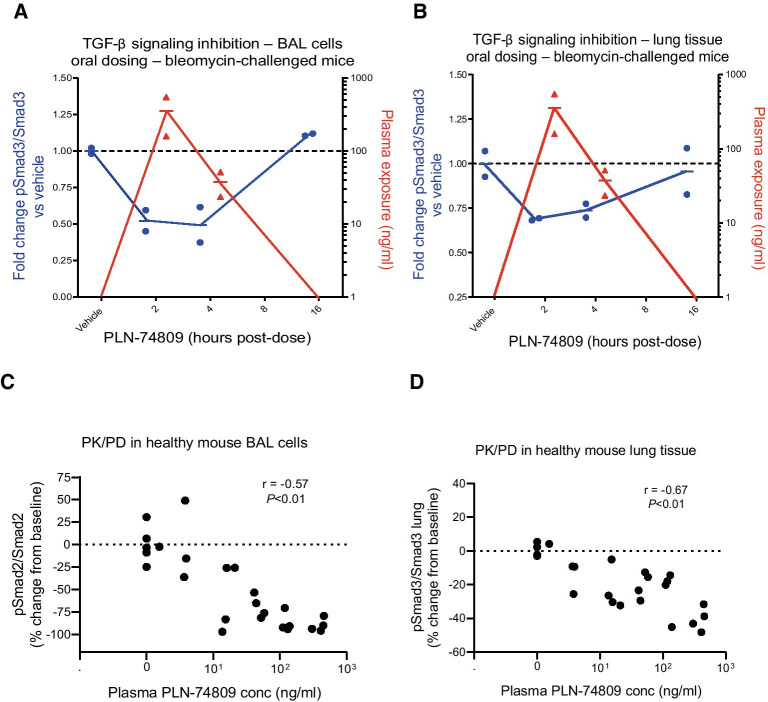
Comparison of plasma PLN-74809 concentrations (red) with pSmad3/Smad3 ratio (blue) in **A** BAL cells and **B** lung tissue from bleomycin-challenged mice. **C** Comparison of plasma PLN-74809 concentrations and pSmad2/Smad2 ratio in BAL cells from healthy mice. **D** Comparison of plasma PLN-74809 concentrations and pSmad3/Smad3 ratio in lung tissue from healthy mice. Bleomycin-challenged mice received three oral, 250 mg/kg doses BID starting 13 days post-challenge, with lung tissue and BAL cells collected 14 days post-challenge at 2, 4, 8, and 16 h post-dose (*n* = 2 per group). Healthy mice received continuous infusion of PLN-74809 (1, 3, 10, 30, or 100 mg/kg/day) via osmotic minipump. BAL: Bronchoalveolar lavage; BID: twice daily; conc: concentration; PD: pharmacodynamics; PK: pharmacokinetics; pSmad2: phosphorylated Smad2; pSmad3: phosphorylated Smad3; TGF-β: transforming growth factor-β

To confirm the pharmacodynamic relationship between PLN-74809 and TGF-β signaling inhibition in the lung, plasma PLN-74809 concentrations were compared with lung tissue and BAL cell Smad2/3 phosphorylation levels in both bleomycin-challenged mice (250 mg/kg BID, oral) and healthy mice (0–100 mg/kg/day, infusion). Cells isolated from BAL reside in the alveolar space and can, therefore, be used to assess TGF-β signaling adjacent to α_v_β_6_-expressing epithelial cells. Phosphorylation of Smad2 or Smad3 in BAL cells and lung tissue from bleomycin-challenged mice (Fig. 5A, B) and healthy mice (Fig. 5C, D) inversely correlated with plasma concentrations of PLN-74809, demonstrating exposure-dependent inhibition of TGF-β activation.

### Comparing the antifibrogenic effects of dual α_v_β_6_/α_v_β_1_ inhibitor, PLN-74809, with nintedanib and pirfenidone

**Fig. 6:**
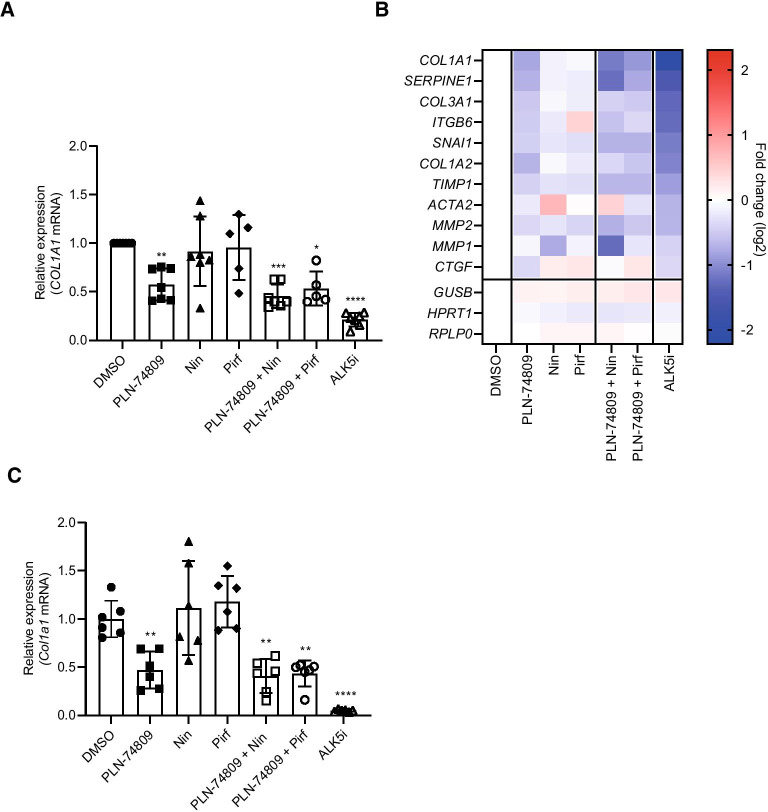
Effect of dual α_v_β_6_/α_v_β_1_ inhibitor (PLN-74809) and clinical standard-of-care drugs (nintedanib and pirfenidone) on **A** *COL1A1* expression and **B** fibrosis-related gene expression in PCLSs prepared from explanted lung tissue from patients with IPF, and **C** *Col1a1* expression in PCLSs prepared from chronic bleomycin-challenged mouse lungs. **A** Data represent mean (± SD) of 5–7 independent IPF tissues with ≥ 3 slices analyzed per patient tissue. Symbols represent results from individual patient tissues. **A**, **B** Treatment effects were normalized to DMSO control for each tissue. Culture and treatment were for 7 days. Concentrations used: PLN-74809 = 200 nM; Nin = 75 nM; Pirf = 50 µM; ALK5i (R 268,712) = 1 µM. **C** Data represent mean (± SD) of a single slice from *n* = 6 mouse lungs. Symbols represent results for individual slices. Treatment effects were normalized to DMSO control. Culture and treatment were performed for 7 days. *P < 0.05 vs DMSO; **P < 0.01 vs DMSO; ***P < 0.001 vs DMSO; ****P < 0.0001 vs DMSO. *ACTA2*: α-smooth muscle actin 2; ALK5i: Activin receptor-like kinase 5 inhibitor; IPF: Idiopathic pulmonary fibrosis; *COL1A1*: Collagen type I alpha I; *COL1A2*: Collagen type I alpha II; *COL3A1*: Collagen type III alpha I; *CTGF*: Connective tissue growth factor; DMSO: dimethyl sulfoxide; *GUSB*: glucuronidase β; *HPRT1*: hypoxanthine phosphoribosyltransferase 1; *ITGB6*: Integrin subunit β 6; *MMP1*: Matrix metalloproteinase 1; *MMP2*: Matrix metalloproteinase 2; mRNA: messenger ribonucleic acid; Nin: Nintedanib; PCLS: Precision-cut lung slice; Pirf: pirfenidone; SD: Standard deviation; *RPLP0*: ribosomal lateral stalk subunit P0; *SERPINE1*: Serpin family E member 1; *SNAI1*: Snail family transcriptional repressor 1; *TIMP1*: tissue inhibitor of metalloproteinase 1

PCLSs prepared from explanted lung tissue from patients with IPF (*n* = 5–7 individual patients) were used to compare the antifibrotic effects of dual α_v_β_6_/α_v_β_1_ inhibitor, PLN-74809, with IPF standard-of-care drugs, nintedanib and pirfenidone. PLN-74809 significantly reduced *COL1A1* mRNA expression when tested alone (42%; *P* < 0.001) or in combination with standard-of-care drugs nintedanib (54%; *P* < 0.0001) or pirfenidone (47%; *P* < 0.001), while nintedanib and pirfenidone alone had no effect on *COL1A1* expression when tested at their approximate clinical maximum observed drug concentration (C_max_) levels of 75 nM and 50 µM, respectively [46] (Fig. 6A). PLN-74809 was also more effective at reducing the expression of additional fibrosis-related genes, relative to vehicle, than nintedanib and pirfenidone, including collagen type III alpha I (*COL3A1*; 30% reduction, *P* < 0.001), (*SERPINE1*; 39% reduction, *P* < 0.001), and tissue inhibitor of metalloproteinase 1 (*TIMP1*; 26% reduction, *P* < 0.05) (Fig. 6B).

PLN-74809, alone or in combination with nintedanib or pirfenidone, also significantly reduced *Col1a1* gene expression in PCLSs prepared from fibrotic mouse lungs following 7 days of treatment (Fig. 6C). Nintedanib and pirfenidone alone did not significantly alter collagen gene expression when tested at their approximate clinical C_max_ levels. The minimum concentrations of PLN-74809, nintedanib, and pirfenidone required to reduce *Col1a1* mRNA levels by 50% in fibrotic mouse lung PCLSs were 172 nM, 897 nM, and 678 µM, respectively, following 3 days of treatment (Additional file 1: Fig. S4).

## Discussion

Previous studies have presented strong evidence for the role of α_v_ integrins in the development and progression of pulmonary fibrosis through their ability to activate latent TGF-β, a key cytokine responsible for fibroblast-to-myofibroblast transition and elevated collagen deposition. α_v_β_6_ inhibition, α_v_β_1_ inhibition, and pan-α_v_ inhibition have each been shown to ameliorate fibrosis in mouse models of IPF [13, 20]. Since cell-to-ECM interactions driven by α_v_ integrins are required for a variety of homeostatic functions throughout the body, the optimal approach for blocking α_v_ integrins in pulmonary fibrosis should be limited to the subset of α_v_ integrins that perpetuate TGF-β activation and fibrogenesis in the fibrotic lung. Here we compared single inhibition of α_v_β_6_ and α_v_β_1_ with dual inhibition of both integrins, demonstrating a clear additive effect from blocking both integrins in reducing collagen expression in ex vivo cultures of lung tissue from patients with IPF. Approximately two-thirds of the TGF-β receptor I kinase-dependent (ALK5-dependent) collagen gene expression in lung explant cultures from patients with IPF could be suppressed by dual α_v_β_6_/α_v_β_1_ inhibition, demonstrating the significant role these two integrins play in driving collagen synthesis in the fibrotic human lung. Furthermore, by avoiding full suppression of TGF-β signaling, dual α_v_β_6_/α_v_β_1_ inhibition may reduce the likelihood of toxicities associated with complete inhibition of the TGF-β pathway. While other α_v_ integrins have been implicated in TGF-β activation, additional experiments performed with mouse PCLSs showed no additional reduction in fibrogenic gene expression resulting from inhibiting additional α_v_ integrins with GSK3008348 or CWHM-12 (i.e. inhibition of α_v_β_3_, α_v_β_5_, and/or α_v_β_8_ in addition to α_v_β_6_ and α_v_β_1_). These data support dual α_v_β_6_/α_v_β_1_ inhibition as the optimal approach for targeting α_v_ integrin-driven lung fibrogenesis.

Positron emission tomography imaging and immunohistochemistry staining techniques have previously shown significantly upregulated levels of α_v_β_6_ in IPF lung tissue [15, 16], however, due to a lack of specific tools for quantification of α_v_β_1_, its relevance in fibrotic human lung disease has remained unclear. Using an electrochemiluminescence assay, we quantified the levels of α_v_β_1_ present in fibrotic and healthy human lung tissue, showing a significant increase in pulmonary α_v_β_1_ levels in patients with IPF. LAP adhesion assays utilizing primary lung fibroblasts and epithelial cells isolated from healthy subjects and/or tissues from patients with IPF were also used to demonstrate the α_v_β_1_- and α_v_β_6_-specific interaction between these cell populations and the latent TGF-β complex, respectively. Epithelial cells and fibroblasts are each known to be key components of the fibroblastic foci central to pulmonary fibrosis. While bronchial epithelial cells were utilized here due to availability, alveolar epithelial cells have specifically been shown to overexpress α_v_β_6_ in IPF [14]. High expression of α_v_β_6_ on aberrant basaloid cells—a recently described population of cells that co-express basal cell, epithelial-to-mesenchymal, and senescence markers unique to IPF lung tissue [47]—may also drive TGF-β activation and fibroblast activity in the fibrotic lung.

PCLSs prepared from tissue of patients with IPF also allowed for direct comparison of the antifibrogenic effects of dual α_v_β_6_/α_v_β_1_ inhibition with standard-of-care drugs, nintedanib and pirfenidone. At concentrations of nintedanib and pirfenidone that approximate C_max_ in patients with IPF, we were unable to detect a significant reduction in collagen gene expression in PCLS cultures. Dose titrations in PCLSs from bleomycin-challenged mouse lungs indicated that a 50% reduction in collagen gene expression required > 10× these concentrations. This result is consistent with previously reported findings that µM and mM concentrations of nintedanib and pirfenidone, respectively, are required for antifibrotic effects in PCLSs [48]. Dual α_v_β_6_/α_v_β_1_ inhibition with PLN-74809, on the other hand, significantly reduced collagen gene expression by ~ 50% at concentrations as low as 2 nM in explanted tissue from a patient with IPF. While having no direct impact on collagen gene expression levels in our IPF PCLS cultures at clinical concentrations, nintedanib and pirfenidone have previously been shown to delay rate of disease progression in patients with IPF, suggesting they may work through alternative mechanisms [49, 50]. Dual α_v_β_6_/α_v_β_1_ inhibition, a therapeutic approach that potently blocks collagen gene expression in tissue from patients with IPF ex vivo may, therefore, have divergent mechanisms of action from standard-of-care drugs, nintedanib and pirfenidone, opening the possibility that combinations of these drugs may provide additive antifibrotic benefits in patients with IPF.

While PCLSs generated from patients with IPF may better replicate some aspects of fibrotic human lung disease than standard mouse models, some obvious drawbacks to this approach remain. Ex vivo culture systems ignore the potential impact of lung-infiltrating cell populations and endocrine factors that may impact disease status or therapeutic efficacy, as well as the pharmacokinetic properties of the inhibitors being tested. The limited duration of IPF PCLS viability combined with the slow progression of native disease (no exogenous fibrogenic factors [e.g. TGF-β or bleomycin] were added to the human PCLS cultures to increase fibrogenesis) also precluded evaluation of drug-induced changes in interstitial collagen protein levels. Our analysis of PCLSs, therefore, focused on markers of TGF-β signaling (Smad phosphorylation) and fibrogenesis (collagen gene expression) indisputably linked to the fibrotic disease pathway, and directly downstream of α_v_ integrin-mediated TGF-β activation. To evaluate the efficacy of dual α_v_β_6_/α_v_β_1_ inhibitor PLN-74809 at blocking accumulation of pulmonary collagen protein we, therefore, relied on the classic in vivo bleomycin mouse model of pulmonary fibrosis. Plasma levels of PLN-74809 were shown to inversely correlate with Smad3 phosphorylation in both pulmonary tissue and BAL cells, confirming inhibition of TGF-β activation in the lung. Oral dosing of PLN-74809 starting 7 days post-bleomycin challenge also resulted in a dose-dependent reduction in the density of interstitial collagen fibrils present in the mouse lungs at 21 days post-bleomycin, as visualized by SHG imaging.

Successful development of TGF-β inhibitors for fibrosis requires an approach that limits inhibition of TGF-β signaling to the site of fibrotic disease. Inhibition of α_v_ integrins offers such an approach, by targeting one mechanism of TGF-β activation enriched in fibrotic tissues without disturbing systemic TGF-β activity. Here we have shown that α_v_β_6_ and α_v_β_1_ represent important targets for reducing TGF-β signaling and fibrogenic gene expression in fibrotic human lung tissue and that the effects of inhibiting these two integrins are additive. We also observed that inhibition of additional α_v_ integrins does not provide additional beneficial antifibrotic effects. These data support the ongoing Phase 2 clinical trials of PLN-74809 examining the effects of dual α_v_β_6_/α_v_β_1_ inhibition in patients with fibrotic lung disease (NCT04396756 and NCT04072315).

## Supplementary Information

**Additional file 1.** Materials and methods. Additional references. **Fig. S1.** (A) Viability of sentinel slices from lung tissue explants on Day 7. (B) Effect of PLN-74809 on Smad2 phosphorylation. (C) Dose titration of PLN-74809 on *COL1A1* expression. **Fig. S2.** Dose titration of PLN-74809 on *Col1a1* expression in PCLSs prepared from bleomycin-challenged mouse lung. **Fig. S3.** Antifibrotic effects of dual α_v_β_6_/α_v_β_1_ inhibition in the bleomycin mouse model. (A) Total lung hydroxyproline content and (B) ^2^H incorporation into lung hydroxyproline in sham-challenged mice and bleomycin-challenged mice treated with vehicle or PLN-74809. **Fig. S4.** Concentration required to decrease *Col1a1* expression by 50% in PCLSs from acute bleomycin-challenged mouse lung. **Table S1.** Donor history for human lung samples. **Table S2.** Custom fibrosis gene panel. **Table S3.** TaqMan primers/probes.

## Data Availability

All data generated or analyzed during this study are included in this published article and its additional information files.

## Abbreviations

Ab: Antibody
ACTA2: α-Smooth muscle actin 2
ALK5: Activin receptor-like kinase 5
ALK5i: ALK5 inhibitor
ANOVA: Analysis of variance
BAL: Bronchioalveolar lavage
BID: Twice daily
Bleo: Bleomycin
C_max_: Maximum observed drug concentration
COL1A1: Collagen type I alpha I
COL1A2: Collagen type I alpha II
COL3A1: Collagen type III alpha I
Conc: Concentration
Cpd A: Compound A
CTGF: Connective tissue growth factor
DMSO: Dimethyl sulfoxide
ECM: Extracellular matrix
GUSB: Glucuronidase β
HLF: Human lung fibroblast
HPRT1: Hypoxanthine phosphoribosyltransferase 1
IC50: 50% Inhibitory concentration
IPF: Idiopathic pulmonary fibrosis
ITGB6: Integrin subunit β 6
LAP: Latency-associated peptide
MMP1: Matrix metalloproteinase 1
MMP2: Matrix metalloproteinase 2
MMP7: Matrix metalloproteinase 7
mRNA: Messenger ribonucleic acid
Nin: Nintedanib
OHP: Hydroxyproline
PBS: Phosphate-buffered saline
PCLS: Precision-cut lung slice
PD: Pharmacodynamics
Pirf: Pirfenidone
PK: Pharmacokinetics
pSmad2: Phosphorylated Smad2
pSmad3: Phosphorylated Smad3
RGD: Arg-Gly-Asp
RPLP0: Ribosomal lateral stalk subunit P0
SD: Standard deviation
SERPINE1: Serpin family E member 1
SHG: Second harmonic generation
SMi: Small-molecule inhibitor
SNAI1: Snail family transcriptional repressor 1
TGF-β: Transforming growth factor-β
TIMP1: Tissue inhibitor of metalloproteinase 1
