# Supplementary material for: Dual inhibition of αvβ6 and αvβ1 reduces fibrogenesis in lung tissue explants from patients with IPF
Source: Respir Res. 2021 Oct 19;22:265. doi: 10.1186/s12931-021-01863-0 (PMC8524858; doi:10.1186/s12931-021-01863-0)
Supplement: Supplementary file 1 — Additional file 1. Materials and methods. Additional references. Fig. S1. (A) Viability of sentinel slices from lung tissue explants on Day 7. (B) Effect of PLN-74809 on Smad2 phosphorylation. (C) Dose titration of PLN-74809 on COL1A1 expression. Fig. S2. Dose titration of PLN-74809 on Col1a1 expression in PCLSs prepared from bleomycin-challenged mouse lung. Fig. S3. Antifibrotic effects of dual αvβ6/αvβ1 inhibition in the bleomycin mouse model. (A) Total lung hydroxyproline content and (B) 2H incorporation into lung hydroxyproline in sham-challenged mice and bleomycin-challenged mice treated with vehicle or PLN-74809. Fig. S4. Concentration required to decrease Col1a1 expression by 50% in PCLSs from acute bleomycin-challenged mouse lung. Table S1. Donor history for human lung samples. Table S2. Custom fibrosis gene panel. Table S3. TaqMan primers/probes. [file 12931_2021_1863_MOESM1_ESM.docx]

Additional file 1

Dual inhibition of α_v_β_6_ and α_v_β_1_ reduces fibrogenesis in lung tissue explants from patients with IPF

Martin L. Decaris*, Johanna R. Schaub*, Chun Chen*, Jacob Cha, Gail G. Lee, Megi Rexhepaj, Steve S. Ho, Vikram Rao, Megan M. Marlow, Prerna Kotak, Erine H. Budi, Lisa Hooi, Jianfeng Wu, Marina Fridlib, Shamra P. Martin, Shaoyi Huang, Ming Chen, Manuel Muñoz, Timothy F. Hom, Paul J. Wolters, Tushar J. Desai, Fernando Rock, Katerina Leftheris, David J. Morgans, Eve-Irene Lepist, Patrick Andre, Eric A. Lefebvre and Scott M. Turner

*Co-first authors

# Additional materials and methods

## Cells and reagents

Primary human lung fibroblasts isolated from normal tissue (NHLF; Lonza CC-2512; Basel, Switzerland) and tissue from patients with idiopathic pulmonary fibrosis (IPF) (dHLF; Lonza CC-7231) were cultured in fibroblast growth medium-2 (Lonza CC-3132) according to manufacturer’s instruction. Primary human lung epithelial cell (normal human bronchial epithelial cells [NHBEs]; Lonza CC-2540) were cultured in bronchial epithelial cell growth medium (BEGM; Lonza CC-4175) according to manufacturer’s instruction. SW480 cells stably transfected with human integrin β_6_ (SW-β_6_) as previously described (E1), Chinese hamster ovary (CHO) cells stably transfected with human integrin α_v_ (CHO-α_v_) as previously described (E2), and mink lung epithelial cells (MLECs) transfected with the firefly luciferase gene driven by the transforming growth factor-β (TGF-β)-inducible plasminogen activator inhibitor-1 promoter as previously described (E3) were cultured in Dulbecco’s Modified Eagle’s Medium (DMEM; Corning #MT10017CV; Corning, NY, USA), supplemented with 10% fetal bovine serum (FBS), penicillin/streptomycin/glutamine (P/S/G; Thermo Fisher #10378016; Waltham, MA, USA), and geneticin (Thermo Fisher #10131027). Precision-cut lung slice (PCLS) tissue culture media consisted of DMEM (Gibco, Thermo Fisher #31053-28), supplemented with P/S/G (Gibco, Thermo Fisher #10378-016). Human PCLS cultures were further supplemented with fungizone (Cytiva #40003.01; Marlborough, MA, USA).

Activin receptor-like kinase 5 inhibitor (ALK5i; R 268712; Tocris; Abingdon, UK), anti-α_v_β_6_ antibody (3G9; Antibody Solutions; Sunnyvale, CA, USA), anti-α_v_ antibody (17E6; EMD Millipore; Burlington, MA, USA), anti-α_v_ antibody (NKI-M9; BioLegend; San Diego, CA, USA), and anti-β_1_ antibody (P5D2; BioLegend) were used as controls in multiple assays. Recombinant integrin proteins α_v_β_1_ (ACROBiosystems #IT1-H52E1; Newark, DE, USA or R&D Systems #6579-AVB; Minneapolis, MN, USA), α_v_β_6_ (ACROBiosystems #IT6-H52E1 or R&D Systems #3817-AV), α_v_β_3_ (R&D Systems #3050‑AV), α_v_β_5_ (R&D Systems #2528-AV), and α_v_β_8_ (R&D Systems #4135-AV) were utilized for integrin ligand-binding assays. Biotin-labeled antibodies against fibronectin (R&D Systems #BAF1918), latency-associated peptide (LAP; R&D Systems #BAF246), and vitronectin (Molecular Innovations; Novi, MI, USA) were utilized for integrin ligand-binding assays. Pan-α_v_ inhibitors, GSK3008348 and CWHM-12, were synthesized based on previously published structures (E4, E5).

## Quantitation of α_v_β_1_ integrin and Smad2/3 phosphorylation levels in lung tissue

For quantitation of integrin α_v_β_1_ protein levels, MSD GOLD 96-well Small Spot Streptavidin plates (Meso Scale Discovery [MSD]; Rockville, MD, USA) were rinsed with phosphate-buffered saline (PBS) with 0.1% Tween-20 (PBST), followed by incubation with biotinylated anti-mouse integrin β_1_ (BioLegend) or anti-human integrin β_1_ (Thermo Fisher) antibodies (1 µg/ml) under agitation for 1 hour (h). A standard curve of recombinant human or mouse integrin α_v_β_1_ was established in PBST. Plates were rinsed again and 25 µL of standard or unknown sample were added in duplicate. Plates were incubated for 1 h, rinsed, and 1 µg/ml of sulfo-tag labeled anti-mouse α_v_ antibody (BioLegend) or anti-human α_v_ antibody (BioLegend) was added, followed by incubation for 1 h under agitation. After a final rinse, 1x read buffer (MSD) was added, and the plate was read using an MSD sector imager instrument (SECTOR S 600).

Biotin-labeled antibodies for total Smad2/3, phosphorylated Smad2 (pSmad2), and total Smad2 (Cell Signaling Technology; Danvers, MA, USA), and antibodies for phosphorylated Smad3 (pSmad3) and total Smad3 (Abcam; Cambridge, UK) were utilized for pSmad electrochemiluminescence assays.

For pSmad3 and total Smad3 quantitation, lung tissues or bronchoalveolar lavage (BAL) cells were lysed in RIPA buffer with protease inhibitor cocktail (Thermo Fisher; #78442). Total protein concentration was normalized across lysates following quantitation by bicinchoninic acid protein assay (Thermo Fisher; #23225). MSD GOLD 96 Small Spot Streptavidin plates were rinsed with PBST, followed by incubation with 1 µg/ml of biotinylated anti-Smad2/3 antibody under agitation for 1 h. Following a second rinse, a 50:50 mix of unknown sample and PBST was added to duplicate wells. Following a 1 h incubation and another rinse, 1 µg/ml of sulfo-tag-labeled anti-pSmad3 or anti-Smad3 antibody was added and incubated for 1 h under agitation. After a final rinse, 1x read buffer was added and the plate was read using an MSD sector imager instrument (SECTOR S 600). pSmad3/Smad3 signal ratios were compared across time points to calculate fold changes.

pSmad2 and total Smad2 quantitation were performed similarly to that described for pSmad3 and total Smad3 above, with the exception that U-PLEX linker conjugated biotinylated anti‑pSmad2 and total Smad2 antibodies were bound to U-PLEX MSD assay plates (MSD; K15227N‑2) according to manufacturer’s instruction, followed by unknown sample incubation and addition of sulfo-tag-labeled anti-Smad2/3 antibody.

## Integrin ligand-binding assays

Microplates were coated with recombinant human integrins dissolved in PBS (2 µg/ml, overnight at room temperature). Following removal of the coating solution, plates were washed with PBS. Plates were blocked with 200 µL/well of blocking buffer (2% bovine serum albumin [BSA] in PBS) at 37 °C for 1 h. Serial dilutions of test compounds and 2 µg/ml of recombinant ligands were added: fibronectin (R&D Systems #1918-Fn) for integrin α_v_β_1_, LAP (Sino Biological #10804-H08H1; Beijing, China) for integrins α_v_β_6_ and α_v_β_8_, or biotin‑tagged vitronectin (Molecular Innovations, Inc. #HVNUBIO) for integrins α_v_β_3_ and α_v_β_5_ in binding/wash buffer (50 mM Tris-HCl, pH 7.5; 0.1% BSA, NaCl 150 mM; 0.02% Tween-20; 1 mM CaCl_2_; 1 mM MnCl_2_; 1 mM MgCl_2_). Plates were incubated for 2 h at 25 °C, washed, and incubated for 1 h with biotin-labeled anti-fibronectin or anti-LAP antibodies (for α_v_β_1_ or α_v_β_6_/α_v_β_8_, respectively). After washing, bound antibody was incubated with peroxidase-conjugated streptavidin (Thermo Fisher #21134; 1:2000 dilution) for 30 minutes (min) at room temperature. Following an additional washing step, 100 μL TMB substrate (BD Biosciences #555214; San Jose, CA, USA) was added and integrin binding was quantified by measuring absorbance at 450 nm by a Tecan Spark 10M plate reader (Tecan; Männedorf, Switzerland). The 50% inhibitory concentration (IC_50_) values for testing compounds were calculated by four-parameter logistic regression using Dotmatics software suite (Dotmatics; Bishop’s Stortford, UK).

## TGF-β co-culture assays

SW-β_6_ cells and MLECs or CHO-α_v_ cells and MLECs were cultured in 96-well plates with test compounds or antibodies for 20 h at 37 °C in a 5% CO_2_ incubator. Small-molecule integrin inhibitors assessed for IC_50_ in blocking integrin-induced TGF-β activation were tested at eight concentrations (1:4 serial dilution, starting at 10 µM or 1 µM, depending on potency). Control reagents analyzed included ALK5i, R-268712, anti-a_v_β_6_ antibody, 3G9 (1 µg/ml), anti-a_v_ antibodies, 17E6 and NKI-M9 (30 µg/ml), and recombinant human TGF-β1 (R&D Systems; 50‑200 pg/ml). Measurement of MLEC luciferase production resulting from α_v_β_6_- or α_v_β_1_-mediated TGF-β activation was performed using the Bright-Glo Luciferase Assay System (Promega; Madison, WI, USA) according to manufacturer’s instruction. Luminescence was quantified by a Tecan Spark 10M plate reader. IC_50_ curves were generated using Dotmatics software suite with five-parameter logistic curve fitting. Geometric mean IC_50_ was calculated for each compound from multiple independent analyses.

## Primary cell LAP adhesion assays

Adhesion assays were performed using the xCELLigence RTCA MP instrument (ACEA Biosciences; San Diego, CA, USA) on 96-well PET plates (ACEA Biosciences; 300600910) via a cell impedance measurement similar to one previously described (E6). Plates were coated with LAP (Sino Biological; 10804-H08H1) overnight at 4 °C and blocked with BSA for 2 h at 37 °C. NHLF (40,000 cells/well), dHLF (40,000 cells/well), or NHBE (20,000 cells per well) in FBS-free media were combined with small-molecule integrin inhibitors or antibodies in the plates and incubated at 37 °C with 5% CO_2_. Small-molecule inhibitors and anti-α_v_β_6_ antibody, 3G9, were assessed for IC_50_ in blocking cell adhesion to LAP at eight concentrations (1:4 serial dilution; final DMSO concentration 0.1%). Anti-α_v_ antibody, 17E6, and anti-β_1_ antibody, P5D2, were tested at a single concentration (10 μg/ml). Cell impedance (cell index) was measured at baseline and every 3 min for up to 24 h using xCELLigence RTCA 2.1.0 Software (ACEA Biosciences). IC_50_ curves were generated and time points calculated to be EC_90_ of cell attachment to LAP (i.e. the time point where cell index reached 90% of maximum in vehicle-treated wells, typically between 1 and 4 h) using five-parameter logistic curve fitting with Prism 8.0.2 (GraphPad). Geometric mean IC_50_ was calculated for each compound following repeat analyses of multiple donor cells.

## PCLS preparation and culture

Human PCLSs were generated from lung tissue explants from patients with IPF undergoing transplant surgery and cultured based on previously reported methods (E7-E12). Slices of tissue (400 µm) were cut using a Compresstome VF-300-0Z (Precisionary Instruments; Natick, MA, USA).

Mouse PCLSs were generated and cultured based on previously reported methods (E13). Briefly, mice were challenged with bleomycin (Hospira; Lake Forest, IL, USA or Teva; Toronto, ON, Canada), either acutely with a single dose (3 U/kg or 4 U/kg) or chronically with multiple doses (3 U/kg bleomycin followed by four 1 U/kg challenges at 2-week intervals). Fourteen days after the final bleomycin challenge, lungs were harvested and 200 μm-thick slices were cut using a Compresstome VF‑300-0Z (Precisionary Instruments).

Slices from each individual core (human) or mouse were distributed among the treatment groups to control for variability in the fibrotic injury within the tissue. All integrin inhibitors were tested at concentrations ≥10x above IC_50_ for blocking latent TGF-β activation determined by *in vitro* assays, unless otherwise noted. After 3 or 7 days in culture, slices were washed and snap‑frozen. Viability of sentinel slices on Days 0/1 and 3/7 was determined using WST-1 Cell Proliferation Reagent (Sigma 5015944001; St Louis, MO, USA).

## PCLS gene expression analysis

For all figures (except Fig. 2B, Fig. S2, and Fig. S4), snap-frozen tissue slices were incubated in one‑third RLT buffer (Qiagen #79216; Venlo, Netherlands) supplemented with 2 mg/ml proteinase K (Sigma-Aldrich #3115887001; St Louis, MO, USA) at 55 °C for 2 h 45 min, with periodic vortexing to generate ribonucleic acid (RNA)-compatible tissue lysate. This lysate was incubated overnight with probes from a custom fibrosis gene panel and NanoString PlexSet reagents (Table S2). The following day, samples were analyzed on a NanoString nCounter SPRINT Profiler (NanoString; Seattle, WA, USA). Messenger RNA (mRNA) counts were normalized to housekeeping genes (*GUSB*, *HPRT1*, *RPLP0*, and/or *POLR1B*) and total mRNA counts, relative to vehicle-treated control from the same experiment, are reported.

For Figs. 2B, S2, and S4, RNA purification and qPCR were performed by Transcriptic, Inc. (Menlo Park, CA, USA) using a proprietary automated protocol. Briefly, RNA was purified on a KingFisher Flex system (Thermo Fisher) and qPCR was performed on a Bio-Rad CFX96 (Bio-Rad; Hercules, CA, USA) with TaqMan primers/probes (Thermo Fisher) listed in Table S3. Ct values were normalized to housekeeping genes (*Rn18sRn45s* and *Hprt*) and reported as ddCT and fold change versus the vehicle-treated samples.

## *In vivo* bleomycin model and tissue collection

Male, 8-week-old C57BL/6 mice (Charles River; Hollister, CA, USA) were administered 3 U/kg bleomycin (Teva) or vehicle (water) via oropharyngeal aspiration while under anesthesia (ketamine/xylazine). PLN-74809 (or vehicle [PBS]) was dosed twice daily, orally (8 h apart) at 100 mg/kg, 250 mg/kg, or 500 mg/kg, beginning 7 days post-bleomycin administration and concluding 21 days post-bleomycin administration. Mice were labeled with stable isotope (^2^H_2_O; Fisher Scientific #AC166300010; Waltham, MA, USA) from 7 to 21 days post-bleomycin challenge via bolus injection (Day 7 post-bleomycin; 5% total body water volume) and drinking water (8% total volume) to incorporate ^2^H into newly synthesized collagen proteins as previously described (E14). Mice were euthanized 2 h after receiving final dose, and plasma (EDTA) and lung tissue collection was performed. Total lung weights were recorded, and right lobes were snap-frozen and stored at ‑80 °C. The left lobe was tied off, insufflated with formalin for 24 h, and transferred to 70% ethanol prior to paraffin embedding. Three mice (two receiving vehicle, one receiving PLN-74809) were euthanized early due to weight loss in excess of 20% resulting from bleomycin challenge and were not included in subsequent analyses.

For pharmacokinetic/pharmacodynamic analysis of dual α_v_β_6_/α_v_β_1_ inhibitor, PLN-74809, in bleomycin-challenged mice, lung tissue and BAL cells were collected 14 days post-bleomycin challenge at 2, 4, 8, and 16 h post-dose. Three 250 mg/kg twice-daily doses were administered orally to mice beginning 13 days post-bleomycin challenge. pSmad3 and Smad3 levels in BAL cells and lung tissue were determined as described above.

For pharmacokinetic/pharmacodynamic analysis of dual α_v_β_6_/α_v_β_1_ inhibitor, PLN-74809, in healthy mice, lung tissue and BAL cells were collected following 7 days of subcutaneous infusion of PLN-74809 (1, 3, 10, 30, and 100 mg/kg) or vehicle via osmotic minipump (ALZET #2001; Cupertino, CA, USA). pSmad2, pSmad3, Smad2, and/or Smad3 levels in BAL cells and lung tissue were determined as described above.

## Measurement of PLN-74809 concentrations in mouse plasma

Plasma concentrations of PLN-74809 (ng/ml) were measured by quantitative LC‑MS/MS, incorporating protein precipitation, extraction, and addition of internal standard (^13^C_6_ isotope of PLN-74809). Chromatographic separations were performed by Shimadzu Nexera HPLC system (Shimadzu; Kyoto, Japan) and Luna Omega C18 50 × 2.1 mm, 2 μM particle size column (Phenomenex; Torrance, CA, USA). Mobile phases composed of water, containing 0.1% formic acid (mobile Phase A) and acetonitrile with 0.1% of formic acid (mobile Phase B), were used in binary flow pumping mode. Flow rate was set at 0.5 ml/min. %B ramped from 10 to 90% in 1 min, 1.5 min at 90%, and 0.5 min equilibrium at 10%. Eluted analytes were detected using Sciex 4000 mass spectrometer (Sciex; Framingham, MA, USA) in positive electrospray mode. PLN-74809 and internal standard were analyzed by multiple reaction monitoring mode. Data were acquired by Analyst 1.6 software (AB Sciex; Foster City, CA, USA). Standards and quality control samples were run concurrently with the unknown samples. Calibration curves were established by plotting peak-area ratios of PLN-74809 to internal standard versus nominal concentration. A weighted least squares linear regression was applied to generate a calibration curve. The calculation of PLN‑74809 concentrations was performed with Analyst 1.6 software (AB Sciex).

## Total hydroxyproline (OHP) and fractional synthesis rate

OHP levels in mouse lung tissue were determined similar to that previously described (E15) using the QuickZyme hydroxyproline assay kit (QuickZyme Biosciences; Leiden, Netherlands). Lung tissue was homogenized in water and hydrolyzed in 6 M HCl (hydrochloric acid, 37%) at 115 °C for 18 h. Hydrolysate was adjusted to 4 M HCl and analyzed for OHP concentrations in a 96-well format, according to manufacturer’s instruction. Absorbance was read at 570 nm with an Infinite M200 PRO microtiter plate reader (Tecan; Crailsheim, Germany) and total lung OHP levels calculated from OHP concentrations (µg OHP/mg tissue) by normalizing to total lung weight. Hydrolysate and plasma were also used to determine fractional synthesis of OHP (% lung OHP synthesized during stable isotope labeling) as previously described (E14).

## Second harmonic generation (SHG) imaging

Formalin-fixed paraffin embedded sections prepared from mouse lung tissue underwent SHG imaging to characterize both the quantity and quality of fibrillar collagen deposition using methods similar to that previously described (E16). Briefly, slides were deparaffinized with xylene, hydrated through a series of ethanol rinses and air-dried. Slides were imaged for SHG (collagen) and TPE (tissue morphology) at 20x magnification via a fully automated non-linear optics imaging system (Genesis 200). SHG and TPE images were analyzed using BisQue ViQi, a cloud-based computing website, using GIS’s proprietary image analysis software for quantitation of total collagen area ratio (total % of area tissue covered by non‑structural fibrillar collagen).

# Additional references

E1. Munger JS, Huang X, Kawakatsu H, Griffiths MJ, Dalton SL, Wu J, Pittet JF, Kaminski N, Garat C, Matthay MA, et al. The integrin alpha v beta 6 binds and activates latent TGF beta 1: a mechanism for regulating pulmonary inflammation and fibrosis. Cell. 1999;96(3):319–28.

E2. Zhang Z, Morla AO, Vuori K, Bauer JS, Juliano RL, Ruoslahti E. The alpha v beta 1 integrin functions as a fibronectin receptor but does not support fibronectin matrix assembly and cell migration on fibronectin. J Cell Biol. 1993;122(1):235–42.

E3. Abe M, Harpel JG, Metz CN, Nunes I, Loskutoff DJ, Rifkin DB. An assay for transforming growth factor‑beta using cells transfected with a plasminogen activator inhibitor-1 promoter‑luciferase construct. Anal Biochem. 1994;216(2):276–84.

E4. Henderson NC, Arnold TD, Katamura Y, Giacomini MM, Rodriguez JD, McCarty JH, Pellicoro A, Raschperger E, Betsholtz C, Ruminski PG, et al. Targeting of αv integrin identifies a core molecular pathway that regulates fibrosis in several organs. Nat Med. 2013;19(12):1617–24.

E5. Procopiou PA, Anderson NA, Barrett J, Barrett TN, Crawford MHJ, Fallon BJ, Hancock AP, Le J, Lemma S, Marshall RP, et al. Discovery of ( S)-3-(3-(3,5-Dimethyl-1 H-pyrazol-1-yl)phenyl)-4-(( R)-3-(2-(5,6,7,8-tetrahydro-1,8-naphthyridin-2-yl)ethyl)pyrrolidin-1-yl)butanoic acid, a nonpeptidic α_v_β_6_ integrin inhibitor for the inhaled treatment of idiopathic pulmonary fibrosis. J Med Chem. 2018;61(18):8417–43.

E6. Wiltshire R, Nelson V, Kho DT, Angel CE, O'Carroll SJ, Graham ES. Regulation of human cerebro‑microvascular endothelial baso-lateral adhesion and barrier function by S1P through dual involvement of S1P1 and S1P2 receptors. Sci Rep. 2016;6:19814.

E7. Bai Y, Krishnamoorthy N, Patel KR, Rosas I, Sanderson MJ, Ai X. Cryopreserved human precision-cut lung slices as a bioassay for live tissue banking. A viability study of bronchodilation with bitter‑taste receptor agonists. Am J Respir Cell Mol Biol. 2016;54(5):656–63.

E8. Mercer PF, Woodcock HV, Eley JD, Platé M, Sulikowski MG, Durrenberger PF, Franklin L, Nanthakumar CB, Man Y, Genovese F, et al. Exploration of a potent PI3 kinase/mTOR inhibitor as a novel anti‑fibrotic agent in IPF. Thorax. 2016;71(8):701–11.

E9. Neuhaus V, Schaudien D, Golovina T, Temann UA, Thompson C, Lippmann T, Bersch C, Pfennig O, Jonigk D, Braubach P, et al. Assessment of long-term cultivated human precision-cut lung slices as an ex vivo system for evaluation of chronic cytotoxicity and functionality. J Occup Med Toxicol. 2017;12:3.

E10. Sanderson MJ. Exploring lung physiology in health and disease with lung slices. Pulm Pharmacol Ther. 2011;24(5):452–65.

E11. Uhl FE, Vierkotten S, Wagner DE, Burgstaller G, Costa R, Koch I, Lindner M, Meiners S, Eickelberg O, Königshoff M. Preclinical validation and imaging of Wnt-induced repair in human 3D lung tissue cultures. Eur Respir J. 2015;46(4):1150–66.

E12. Westra IM, Pham BT, Groothuis GMM, Olinga P. Evaluation of fibrosis in precision-cut tissue slices. Xenobiotica. 2013;43(1):98–112.

E13. Lyons-Cohen MR, Thomas SY, Cook DN, Nakano H. Precision-cut mouse lung slices to visualize live pulmonary dendritic cells. J Vis Exp. 2017;(122):55465.

E14. Decaris ML, Gatmaitan M, FlorCruz S, Luo F, Li K, Holmes WE, Hellerstein MK, Turner SM, Emson CL. Proteomic analysis of altered extracellular matrix turnover in bleomycin-induced pulmonary fibrosis. Mol Cell Proteomics. 2014;13(7):1741–52.

E15. Woessner JF. The determination of hydroxyproline in tissue and protein samples containing small proportions of this imino acid. Arch Biochem Biophys. 1961;93:440–7.

E16. Liu F, Zhao JM, Rao HY, Yu WM, Zhang W, Theise ND, Wee A, Wei L. Second harmonic generation reveals subtle fibrosis differences in adult and pediatric nonalcoholic fatty liver disease. Am J Clin Pathol*.* 2017;148(6):50–512.

### Additional figures

**Fig. S1** (A) Viability of sentinel slices from lung tissue explants on Day 7. (B) Effect of PLN‑74809 on Smad2 phosphorylation in PCLSs from IPF explant tissue. (C) Dose titration of PLN-74809 on *COL1A1* expression in PCLSs from IPF explant tissue.

**A B**

**C**

(A) Lung tissue explants from *n* = 7 patients. (B) Data represent mean (± SD) of 3 independent IPF tissues with treatment effects normalized to DMSO control for each tissue. Symbols represent results for individual patients. (C) Data are mean (± SD) of *n* = 6 slices. Treatment effects were normalized to DMSO control. Symbols represent results for individual slices. ALK5i R 268712 = 1 µM

****P*<0.001 vs DMSO; *****P*<0.0001 vs DMSO

ALK5i: Activin receptor-like kinase 5 inhibitor; *COL1A1*: Collagen type I alpha I; DMSO: Dimethylsulfoxide; IPF: Idiopathic pulmonary fibrosis; mRNA: Messenger ribonucleic acid; PCLS: Precision-cut lung slice; pSmad2: Phosphorylated Smad2; SD: Standard deviation

**Fig. S2** Dose titration of PLN-74809 on *Col1a1* expression in PCLSs prepared from bleomycin-challenged mouse lung


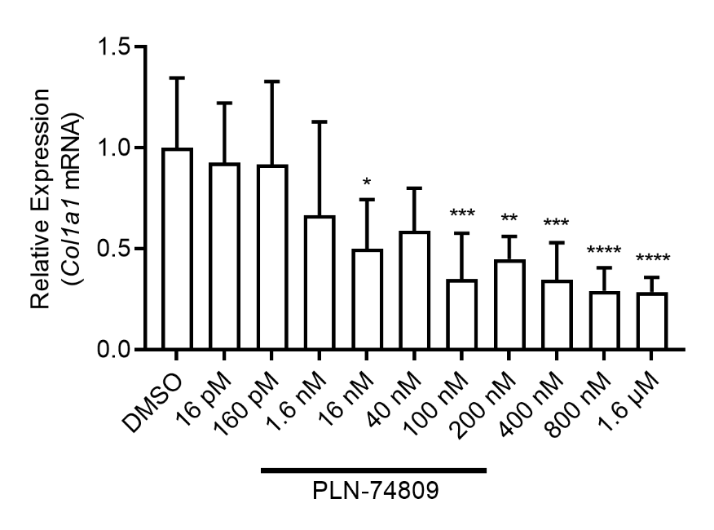


Data are mean (± SD) of a single slice from *n* = 5 mouse lungs. Culture and treatment were for 3 days. Treatment effects were normalized to DMSO control
**P*<0.05 vs DMSO; ***P*<0.01 vs DMSO; ****P*<0.001 vs DMSO; *****P*<0.0001 vs DMSO

*Col1a1*: Collagen type I alpha I; DMSO: Dimethylsulfoxide; mRNA: Messenger ribonucleic acid; PCLS: Precision-cut lung slice; SD: Standard deviation

**Fig. S3** Antifibrotic effects of dual α_v_β_6_/α_v_β_1_ inhibition in the bleomycin mouse model.
(A) Total lung hydroxyproline content and (B) ^2^H incorporation into lung hydroxyproline in sham-challenged mice and bleomycin-challenged mice treated with vehicle or PLN-74809.

**A B**

Mice received dual α_v_β_6_/α_v_β_1_ inhibitor PLN-74809 (dosed orally 100–500 mg/kg twice a day) or vehicle (PBS) from 7 to 21 days post-bleomycin-induced lung challenge (*n* = 10–15 mice per group). Lung tissue from a subset of mice labeled with ^2^H_2_O from 7 to 21 days post-bleomycin-induced lung challenge was evaluated for fraction of newly deposited OHP. Data presented as box and whisker plots with minimum, 25^th^, 50^th^, 75^th^ percentile and maximum values indicated

**P*<0.05 vs vehicle; ***P*<0.01 vs vehicle

bleo: Bleomycin; OHP: Hydroxyproline; PBS: Phosphate-buffered saline

**Fig. S4** Concentration required to decrease *Col1a1* expression by 50% in PCLSs from acute bleomycin-challenged mouse lung


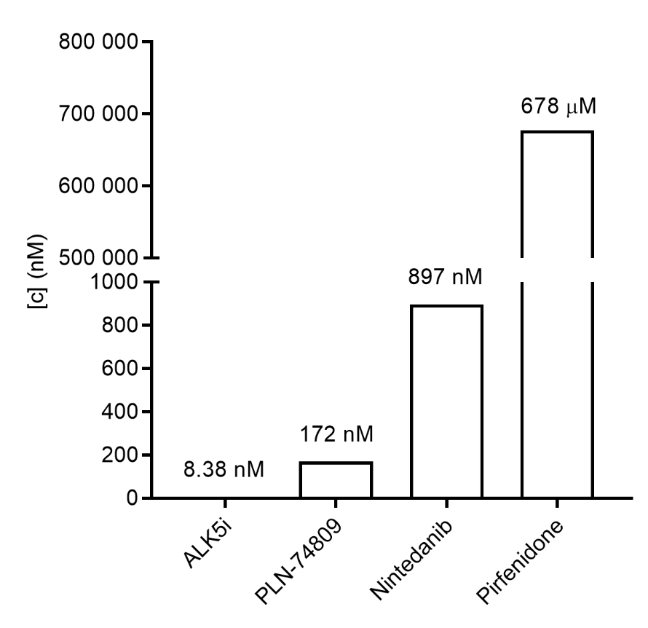


Data were calculated by extrapolation from a dose–response curve generated by combining data from multiple independent experiments (ALK5i *n* = 11, PLN-74809 *n* = 9, nintedanib *n* = 3, pirfenidone *n* = 3) across a range of inhibitor concentrations. Culture and treatment were for 3 days

ALK5i: Activin receptor-like kinase 5 inhibitor; c: Concentration; *Col1a1*: Collagen type I alpha I; PCLS: Precision-cut lung slice

### Additional tables

**Table S1** Donor history for human lung samples

| **Study** | **Diagnosis** | **Sex** | **Age**  **(mean years ± SD)** | **Pretransplant therapy** |
| --- | --- | --- | --- | --- |
| PCLS (gene) | 7 IPF, 1 probable IPF | 7M/1F | 62 ± 4 | 5 Nin, 2 Pirf |
| PCLS (pSmad2) | 3 IPF | 3M | 64 ± 6 | 1 Nin, 1 Pirf |
| α_v_β_1_ quantitation | 21 IPF | 14M/7F | 64 ± 6 | 6 Nin, 6 Pirf, 3 Nin + Pirf, 1 Unk |
|  | 10 normal (rejected donor) | 6M/4F | 48 ± 21 | N/A |

F: Female; IPF: Idiopathic pulmonary fibrosis; M: Male; N/A: Not applicable; Nin: Nintedanib; PCLS: Precision-cut lung slice; Pirf: Pirfenidone; pSmad2: Phosphorylated Smad2; SD: Standard deviation; Unk: Unknown

**Table S2** Custom fibrosis gene panel

| **Gene** | **Accession** | **Position** |
| --- | --- | --- |
| *ACTA2* | NM_001613.1 | 646-745 |
| *COL1A1* | NM_000088.3 | 5211-5310 |
| *COL1A2* | NM_000089.3 | 2636-2735 |
| *COL3A1* | NM_000090.3 | 181-280 |
| *CTGF* | NM_001901.2 | 1101-1200 |
| *GUSB* | NM_000181.3 | 1900-1999 |
| *HPRT1* | NM_000194.1 | 241-340 |
| *ITGB6* | NM_000888.3 | 1091-1190 |
| *MMP1* | NM_002421.3 | 1118-1217 |
| *MMP2* | NM_004530.5 | 1698-1797 |
| *MMP7* | NM_002423.3 | 312-411 |
| *RPLP0* | NM_001002.3 | 251-350 |
| *SERPINE1* | NM_000602.2 | 2471-2570 |
| *SNAI1* | NM_005985.2 | 64-163 |
| *TIMP1* | NM_003254.2 | 330-429 |

**Table S3** TaqMan primers/probes

| **Gene** | **Accession** | **Assay ID** |
| --- | --- | --- |
| *Col1a1* | NM_007742.3 | Mm00801666_g1 |
| *Hprt* | NM_013556.2 | Mm03024075_m1 |
| *Rn18s, Rn45s* | NR_003278.3 | Mm03928990_g1 |
